# Supplementary material for: Diversity and Functional Potential of Gut Bacteria Associated with the Insect Arsenura armida (Lepidoptera: Saturniidae)
Source: Insects. 2025 Jul 10;16(7):711. doi: 10.3390/insects16070711 (PMC12295806; doi:10.3390/insects16070711)
Supplement: Supplementary file 1 [file insects-16-00711-s001.zip › insects-3603014-supplementary.pdf]

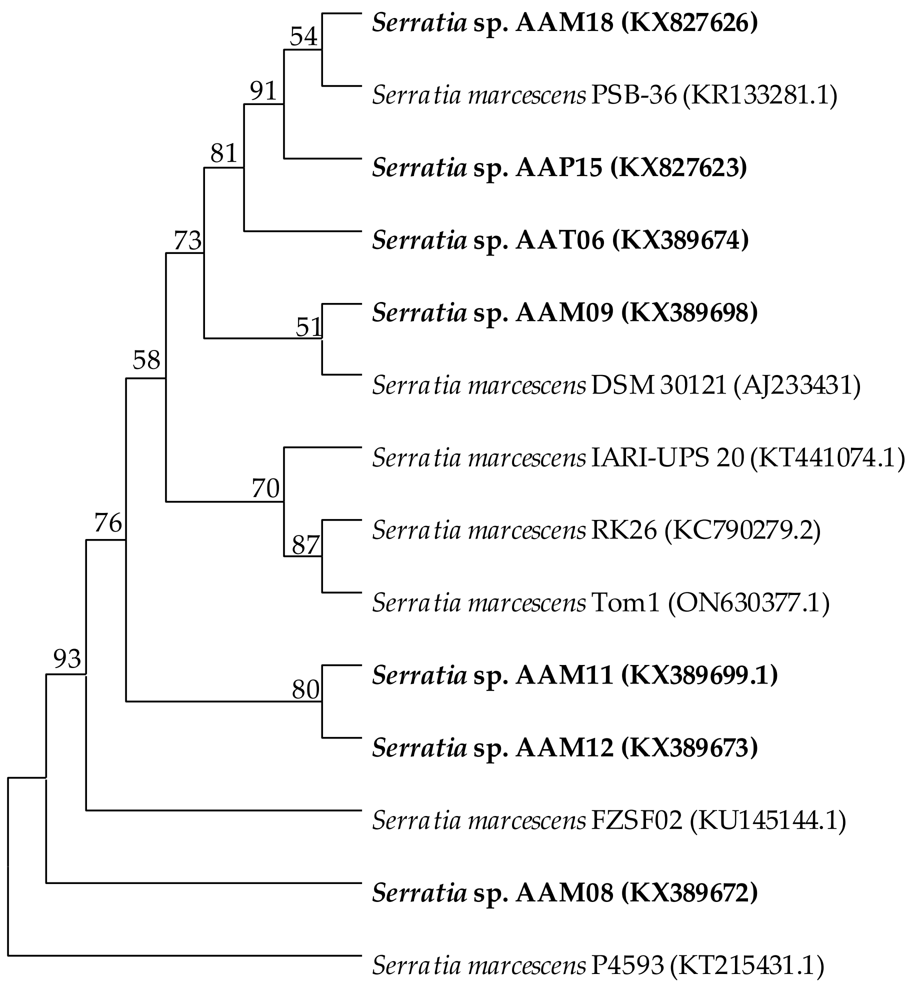

Figure S1. Phylogenetic tree base on the 16S *r*RNA gene of bacterial strains grouped within the genus *Serratia* isolated from gut of *Arsenura armida* constructed using Neighbor-Joining method.

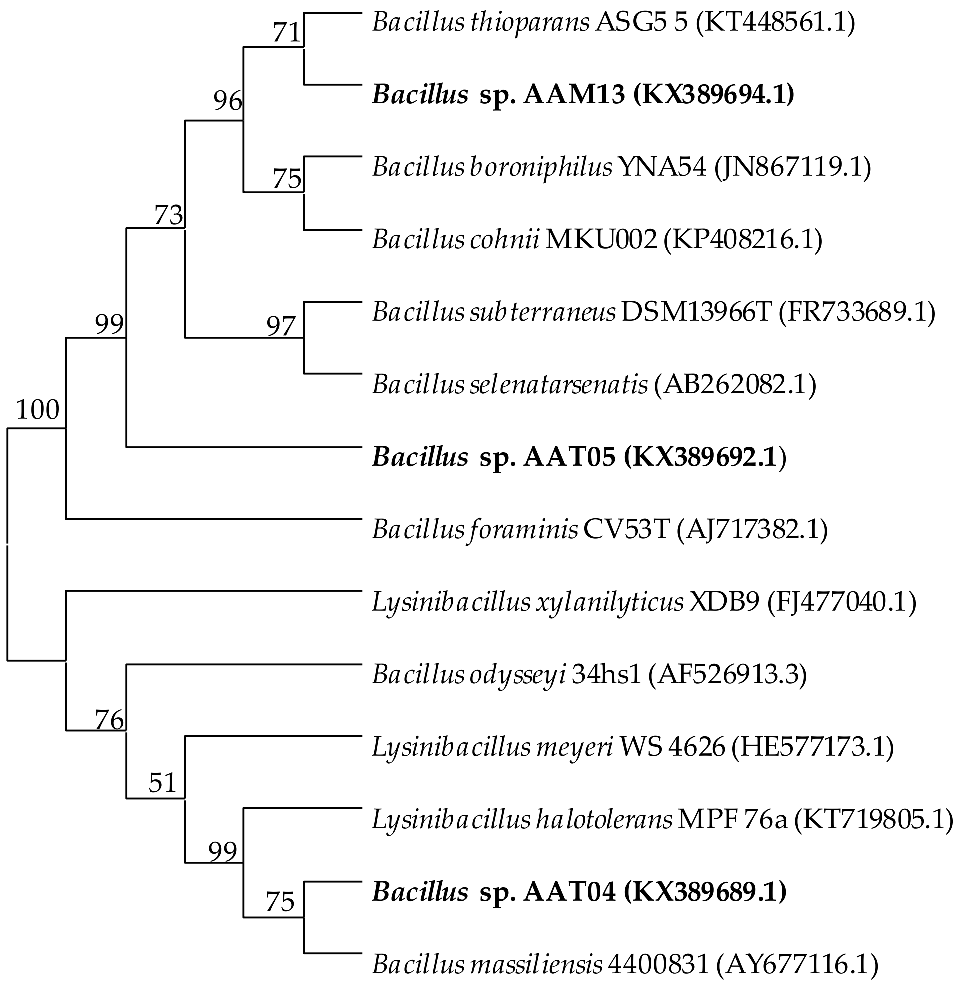

Figure S2. Neighbor-Joining phylogenetic tree based on the 16S *r*RNA gene sequences of bacterial strains belonging to the genus *Bacillus*, isolated from the gut of *Arsenura armida*.
